# Cascade Catalysis for CO_2_ Upgrading to Long-Chain Products

**DOI:** 10.1021/acscentsci.5c02358

**Published:** 2026-07-17

**Authors:** Yutong Wang, Min Kuang, Gengfeng Zheng

**Affiliations:** † Laboratory of Advanced Materials, Shanghai Key Laboratory of Molecular Catalysis and Innovative Materials, Shanghai Academy of Natural Sciences, 12478 Fudan University, Shanghai, 200438, China; ‡ State Key Laboratory of Advanced Fiber Materials, College of Materials Science and Engineering, 12475 Donghua University, Shanghai 201620, China

## Abstract

Cascade catalytic conversion of CO_2_ has recently emerged as a transformative strategy for multilevel CO_2_ valorization. By integration of electrocatalytic CO_2_ reduction with thermocatalytic and/or biocatalytic processes, this approach enables stepwise construction of complex products from simple carbon feedstocks. The electrocatalytic stage supplies key intermediates or upstream products such as CO, formate, formaldehyde, or ethanol, which can be further converted into value-added multicarbon products including sugars, polymers, and other fine chemicals through downstream coupling. Recent demonstrations, ranging from CO_2_-to-sugar and CO_2_-to-polyketone conversions to artificial CO_2_-to-polymer biocatalytic pathways, have demonstrated the great potential of cascade strategies in bridging electro-, thermo-, and biochemistry. This Outlook discusses recent progress in electrochemical cascade CO_2_ conversion and outlines the key design goals for this field, including full-cascade performances, coupled catalyst-reaction optimizations, mechanistic understanding across complex interfaces, and integrated reactor architectures.

## Graphical abstract

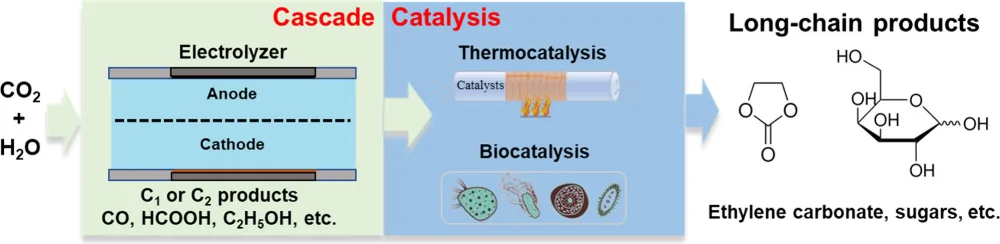

## 1 Introduction

The electrocatalytic carbon dioxide reduction reaction (eCO_2_RR) represents a fast-developing and promising strategy to mitigate the ever-rising atmospheric CO_2_ levels while producing value-added fuels and chemical feedstocks.^1–4^ By coupling electricity from renewable sources with catalytic transformation, the eCO_2_RR suggests a sustainable route to store intermittent energy in chemical bonds. Over the past decade, significant advances in catalyst designs, mechanistic understanding, and reactor engineering have enabled efficient conversion of CO_2_ into C_1_, C_2_, and C_3+_ products such as CO, formate, methane, ethylene, ethanol, and *n*-propanol.^5–9^ Despite those achievements, the direct electrochemical synthesis of more complex products, such as sugars, long-chain alcohols, or polymers, remains challenging. The difficulty arises from the complexity of multielectron and multiproton transfer steps and the instability of key intermediates under electrochemical conditions.

To overcome these intrinsic limitations, the cascade catalytic reaction of CO_2_ has recently emerged as a promising strategy for multilevel CO_2_ valorization. In a typical cascade catalytic process, CO_2_ is first electrochemically reduced into reactive intermediates or upstream products, such as CO, formaldehyde, glycolaldehyde, acetate, or ethanol.^10–13^ Those chemicals then undergo electrocatalytic, thermocatalytic, and/or biocatalytic transformations in a cascade modular manner to form more complex molecules, allowing one to connect distinct catalytic steps into an integrated reaction network.^14,15^ For example, in electrocatalytic-thermocatalytic systems, the tandem CO_2_-to-CO/ethylene conversion followed by thermocatalytic copolymerization has been demonstrated to produce polyketones, and the electrogenerated products such as formaldehyde and glycolaldehyde have been utilized in Formose reactions to yield higher-order sugars.^10,16^ In electrocatalytic-biocatalytic systems, integrating CO_2_ electroreduction with enzyme/microbial fermentation can enable the CO_2_-to-glucose or CO_2_-to-polymer conversions under mild, biocompatible conditions.^17,18^ Several representative examples are presented in Table 1, which also compares several criteria including whether CO_2_ serves as the sole carbon source, whether products with carbon chain lengths of six or greater can be obtained, and long-term operational stability.

**Table 1:** Representative Examples of Cascade Catalysis for CO_2_ Upgrading to Long-Chain Products

cascade system	CO_2_RR reaction condition and product (FE)	downstream reaction condition	final product	yield	CO_2_ as the only C source	C_*n*_ (*n* ≥ 6)	stability	ref
Electrochemical cascade	oxide-derived Cu	0.1 M KBr for anode, 0.1 M KNO_2_ for Cathode	Triethanolamine	N.A.	Yes	Yes	8 h	^24^
	1 M KOH							
	C_2_H_4_ (37%)							
								
	copper-sputtered PTFE	WO_3_ nanoarrays	Ethylene carbonate	1.288 mmol h^–1^	Yes	No	10 h	^1^
	0.1 M KHCO_3_	0.5 M KBr						
	C_2_H_4_ (51%)							
								
	Cu/Au heterojunction	Oxygen-functionalized graphite flake	1,1-Diethoxyethane	0.35 mmol cm^–2^ h^–1^	Yes	Yes	10 h	^25^
	1 M KOH							
	CH_3_CH_2_OH (61%)	0.5 M H_2_SO_4_						
Electro-thermocatalytic cascade	Cu nanoparticles	75 °C, excess Ca(OH)_2_, pH 11	Sugars	2.6 μmol	No	Yes	N.A.	^11^
	0.1 M KHCO_3_							
	Glycolaldehyde (2–3%)							
								
	HD-copper	80 °C, Rh-DPPB catalyst	Propionaldehyde	0.77 mmol	Yes	No	30 h	^20^
	0.1 M KHCO_3_							
	C_2_H_4_, CO, H_2_							
								
	Cu/C	160 °C, Rh_1_Co_3_/MCM-41 catalyst	C_3_ oxygenates	11.8 μmol min^–1^	Yes	No	N.A.	^29^
	0.05 M KHCO_3_							
	C_2_H_4_, CO, H_2_							
								
	Cu and Ag GDE	25 °C, Pd-PP catalyst	Polyketone	57.9 mg	Yes	Yes	1–2 h	^10^
	1 M KHCO_3_							
	C_2_H_4_, CO							
								
	Pd/C	370–450 °C, FeCo alloy	Carbon nanofibers	2.5 g g_metals_^–1^ h^–1^	Yes	Yes	11 h	^31^
	0.05 M KHCO_3_							
	CO, H_2_							
								
	W-CuO_*x*_	TS-1, stirring for 12 h	Ethylene oxide	422.3 μmol h^–1^	Yes	No	60 h	^33^
	2 M K_2_CO_3_							
	C_2_H_4_,(66.9%)							
								
	Cu	600 °C, gallium- and phosphorus-modified zeolite ZSM-5 catalyst	Benzene, toluene, ethylbenzene, and xylene isomers	N.A.	Yes	Yes	3 h	^34^
	C_2_H_4_ (23.1%)							
Electro-biocatalytic cascade	Bi nanowires porous solid-electrolyte Formate (>80%)	Five-enzyme cascade platform	l-Sorbose	105.0 mg L^–1^h^–1^	Yes	Yes	120 h	^16^
								
	Cu–Ag	*C. necator*	Poly(3-hydroxybutyrate)	32 ± 3.5 mg L^–1^ h^–1^	Yes	Yes	8 h	^17^
	0.1 M KH_2_PO_4_							
	C_2_ oxygenate (35%-39%)							
								
	Ni–N–C single atom grain-boundary rich Cu solid-electrolyte CO (nearly 100%)	Genetically engineer *Saccharomyces cerevisiae*	Glucose	8.90 μmol per gram yeast per hour	Yes	Yes	140 h	^41^
	CH_3_COOH (46%)							

Compared with conventional single-step eCO_2_RR, the cascade catalytic strategy presents several unique advantages. First, it provides high flexibility in catalyst and process design, enabling integration of heterogeneous, homogeneous, and biological catalysis under their respective optimal environments and thus is capable of promoting carbon utilization and energy efficiency. Second, it enhances scalability and compatibility with existing industrial processes, bridging electrochemical synthesis with thermochemical upgrading and microbial fermentation. More importantly, it opens new opportunities for generating higher-order carbon products such as sugars, polymers, and fine chemicals. These advances collectively signify a paradigm shift from isolated electrocatalysis toward reaction networks that integrate electrochemical, thermal, and biological catalysis. As a result, the cascade catalytic design suggests an artificial carbon conversion and upgrading strategy that emulates biological metabolism in a controllable, sustainable manner. By combining the strengths of different catalytic approaches, cascade electrocatalytic conversion can bring substantial opportunities for sustainable, programmable, and high-value CO_2_ utilization (Figure 1). In this Outlook, we will highlight recent progress in this emerging field, outline current challenges, and discuss future opportunities for achieving carbon-neutral chemical manufacturing via intelligent reaction coupling and interface design.

**Figure 1:**
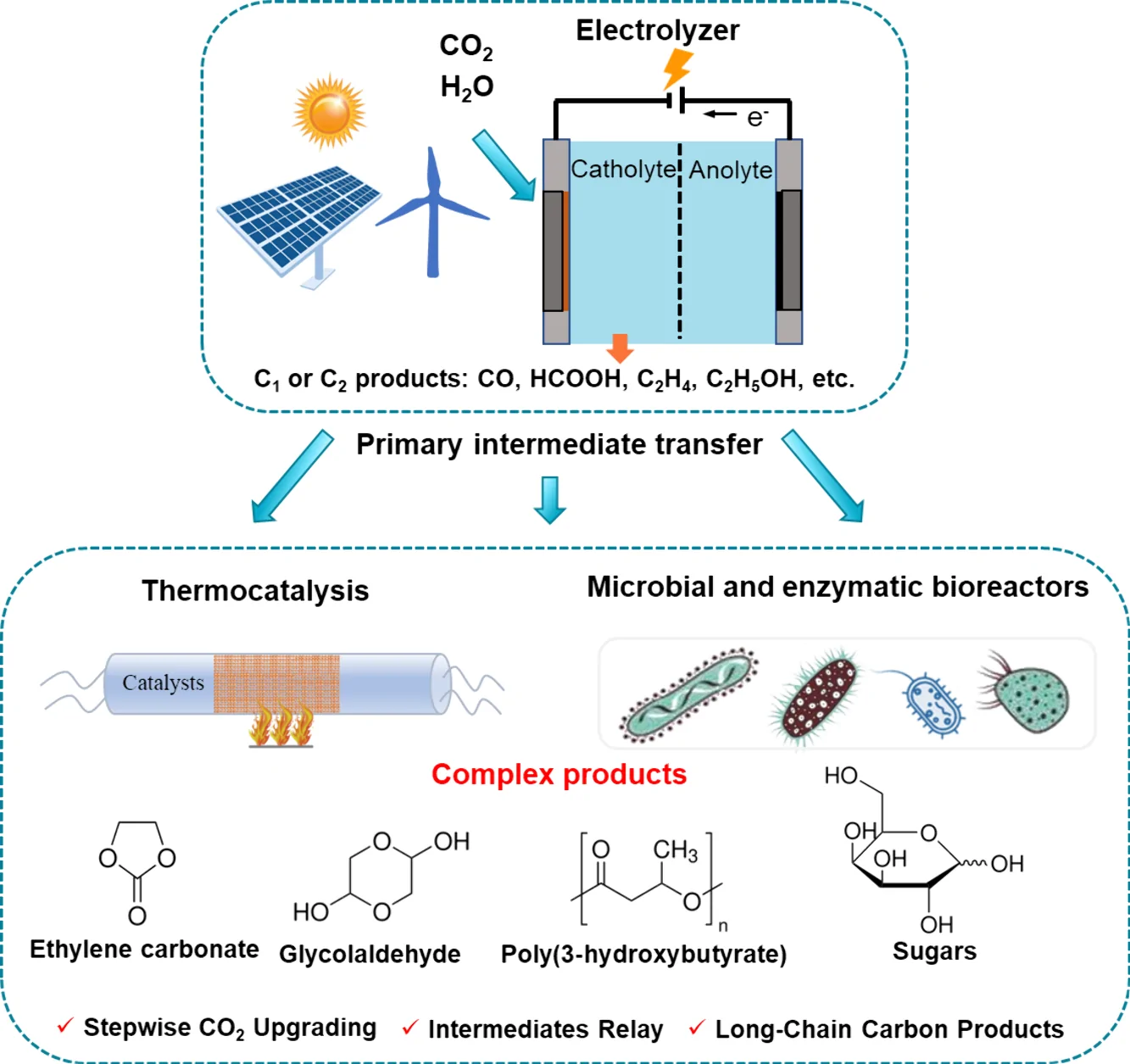
Cascade pathways for artificial CO_2_ valorization. The schematic illustrates the integrated cascade electrocatalytic strategy for multilevel CO_2_ conversion. In the first stage, electrocatalytic CO_2_ reduction powered by renewable electricity generates key first-level products such as CO, formate, formaldehyde, glycolaldehyde, and ethanol. Those intermediates are subsequently directed into thermocatalytic or biocatalytic processes to construct higher-order carbon frameworks, including sugars, polymers, and fine chemicals. The modular coupling between electrocatalytic, thermochemical, and biological reactions can enable stepwise molecular upgrading with independent optimization of each reaction environment. This cascade architecture bridges distinct catalytic regimes, enhances overall carbon utilization, and provides a sustainable route toward artificial CO_2_ valorization and circular chemical manufacturing.

By combining the strengths of different catalytic approaches, cascade electrocatalytic conversion can bring substantial opportunities for sustainable, programmable, and high-value CO_2_ utilization.

## 2 Cascade Catalytic Reactions of CO_2_

Cascade catalytic reactions of CO_2_ generally refer to the feeding of electrocatalytic CO_2_ reduction products into subsequent electrocatalytic, thermocatalytic, or biocatalytic reactors/processes, either without or with feeding additional reactants, thus achieving greater CO_2_ valorization by producing value-added products.^14,19–21^ This upgrading strategy relies on deep insights into CO_2_ activation using advanced surface-sensitive techniques on the atomic scale. For instance, Frei and co-workers reported the activation of CO_2_ to CO on Ni single-atom catalysts, where the CO formation rate evolved linearly with the amount of atomic-dispersed Ni.^22^ Utilizing ambient-pressure-based surface techniques such as scanning tunneling microscopy and X-ray photoelectron spectroscopy, the Park group investigated the step-broken Cu nanocluster evolution on the Cu(997) surface under CO_2_ and revealed the influence of surface morphology structures at the atomic level.^23^ Just as the electrochemical CO_2_RR has resulted in substantial progress in generating C_1–3_ products, the cascade catalytic CO_2_ conversion can reveal new prospects for providing more value-added products, as well as new opportunities for a carbon-neutral chemical manufacturing industry.

### 2.1 Electrocatalytic-Electrocatalytic Cascade Reactions for CO_2_ Conversion

Although the electrochemical conversion of CO_2_ to C_1–3_ products has been widely reported with substantial improvements in the past several years, only limited longer carbon chain (C_4+_) compounds have been directly synthesized from the eCO_2_RR with appreciable selectivities and activities. Facing this challenge, constructing a multistaged electrocatalytic-electrocatalytic cascade reaction system may relieve the limitation and provide the opportunity of achieving more products using CO_2_ as the sole carbon source. For instance, Zheng and co-workers demonstrated the capability of upgrading CO_2_ and nitrite into triethanolamine, where two tandem electrolysis cells were designed (Figure 2a,b).^24^ The first electrolyzer was used for electroconversion of CO_2_ into C_2_H_4_ with an oxide-derived Cu as the cathode catalyst, and the *in situ* generated C_2_H_4_ was delivered to the second electrolyzer, in which Br^–^ anion was electrochemically oxidized in the anode to form Br_2_ and then reacted with C_2_H_4_ to produce bromoethanol. The cathode reaction in the second reactor was a nitrite reduction reaction to form ammonia, which further reacted with bromoethanol to generate the final product triethanolamine. Similarly, the Li group reported a cascade electrocatalytic route for the production of ethylene carbonate (Figure 2c).^1^ This approach also started with eCO_2_RR to C_2_H_4_ in the first electrolyzer, and an electrochemical oxidation of Br^–^ to Br_2_ at the anode reacted with C_2_H_4_ to produce bromoethanol in the second electrolyzer, followed by a catalytic reaction between bromoethanol and CO_2_ to form ethylene carbonate. Ma and co-workers reported a tandem electrocatalytic strategy for synthesizing 1,1-diethoxyethane (DEE), where CO_2_ was first electrochemically reduced to ethanol, then selectively electrooxidized and acetalized to form DEE (Figure 2d).^25^ Those aforementioned studies suggest the potential of electrocatalytic-electrocatalytic cascade system for valorizing CO_2_ to more compounds, although the obtained carbon chain lengths are still limited.

**Figure 2:**
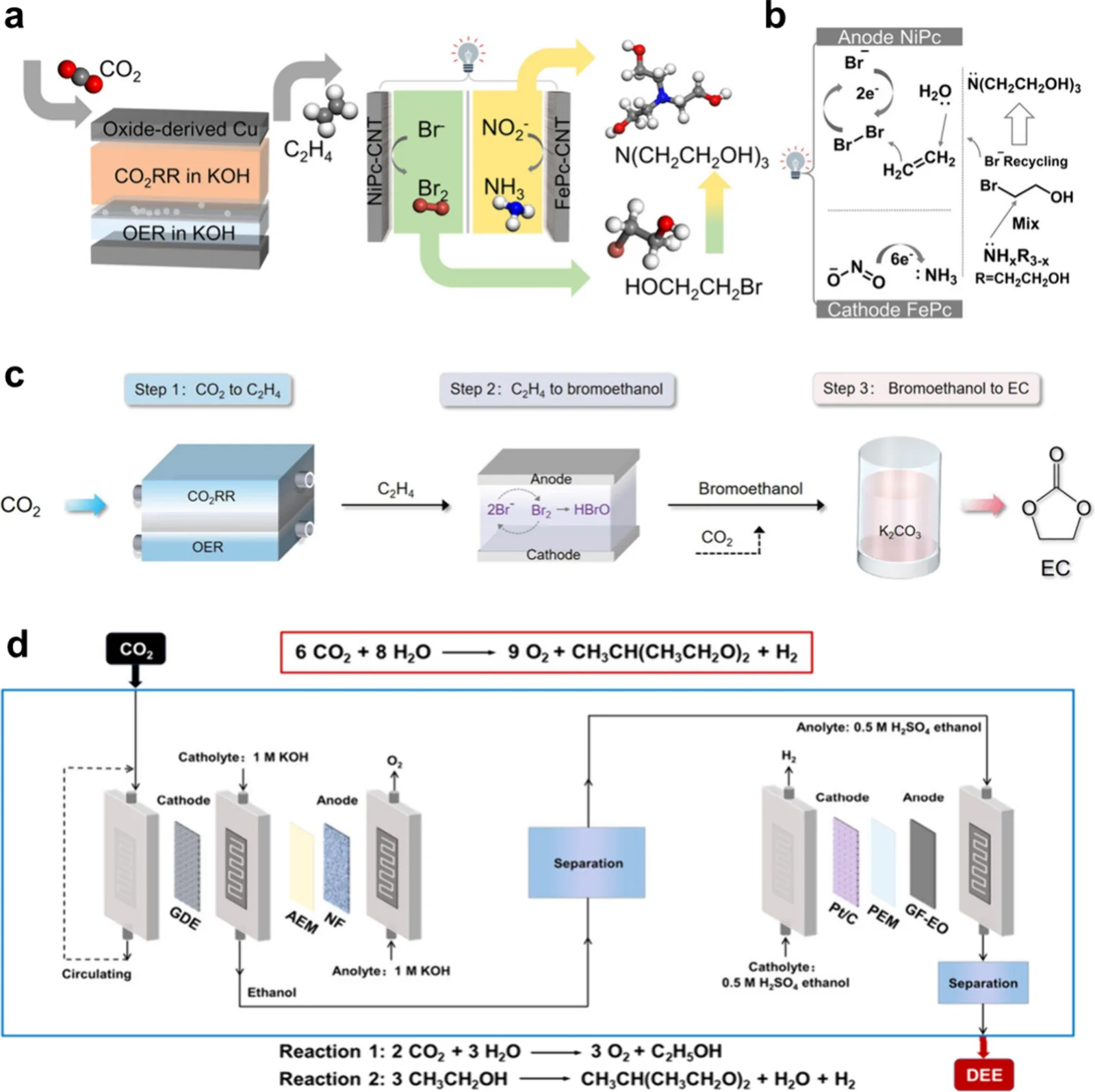
Electrocatalytic-electrocatalytic cascade reaction for CO_2_ conversion. (a) Scheme showing electrocatalytic generation of triethanolamine using CO_2_, nitrite, and water by two tandem electrolyzers. (b) The reaction process in the second reactor, where a NiPc electrocatalyst was used at the anode for C_2_H_4_ electrocatalytic oxidation to bromoethanol and FePc was used at the cathode for NH_3_ production. Adapted with permission from ref 24. Copyright 2023 Wiley-VCH GmbH. (c) Schematic showing the three-step cascade catalysis, which couples CO_2_ to ethylene, Br-assisted C_2_H_4_ to bromoethanol and bromoethanol with CO_2_ to ethylene carbonate (EC). Reproduced from ref 1. Copyright 2024 American Chemical Society. (d) Schematic diagram of the synthesis of DEE from CO_2_ by a tandem electrocatalytic route. Reproduced from ref 25. Copyright 2025 American Chemical Society.

### 2.2 Electrocatalytic-Thermocatalytic Cascade Reactions for CO_2_ Conversion

The cascade electrocatalytic-thermocatalytic process shows broad application prospects and potential in artificial CO_2_ valorization to value-added chemicals. For example, it is possible to develop an abiotic CO_2_-to-sugar conversion that only requires H_2_O, CO_2_, and renewable energy, which can be widely utilized for food production, fine chemical engineering, and fuel generation. Formaldehyde and glycolaldehyde, produced by eCO_2_RR, can be considered as the feedstocks in the Formose reaction for sugar production,^26^ and the feasibility of the CO_2_-to-sugar cascade catalytic conversion has been recently reported.^27,28^ For example, Yang and co-workers demonstrated an electrocatalytic route to produce formaldehyde and glycolaldehyde from CO_2_, which was integrated with a thermocatalytic process via the Formose reaction to form sugars such as glucose.^11^ In this work, a Cu nanoparticle electrocatalyst was first applied for the CO_2_-to-glycolaldehyde conversion, and then glycolaldehyde was used as the reactant in the Formose reaction (Figure 3a). Shao and co-workers demonstrated that the electrochemical CO_2_-to-glycolaldehyde conversion and subsequent coupling can produce C_4_ carbohydrates (e.g., tetroses).^27^ Although the proof-of-concept of this approach has been verified, the glycolaldehyde levels directly generated from CO_2_ in those studies were generally not sufficient enough to sustain the subsequent tandem electrochemical conversion to sugars or carbohydrates.

**Figure 3:**
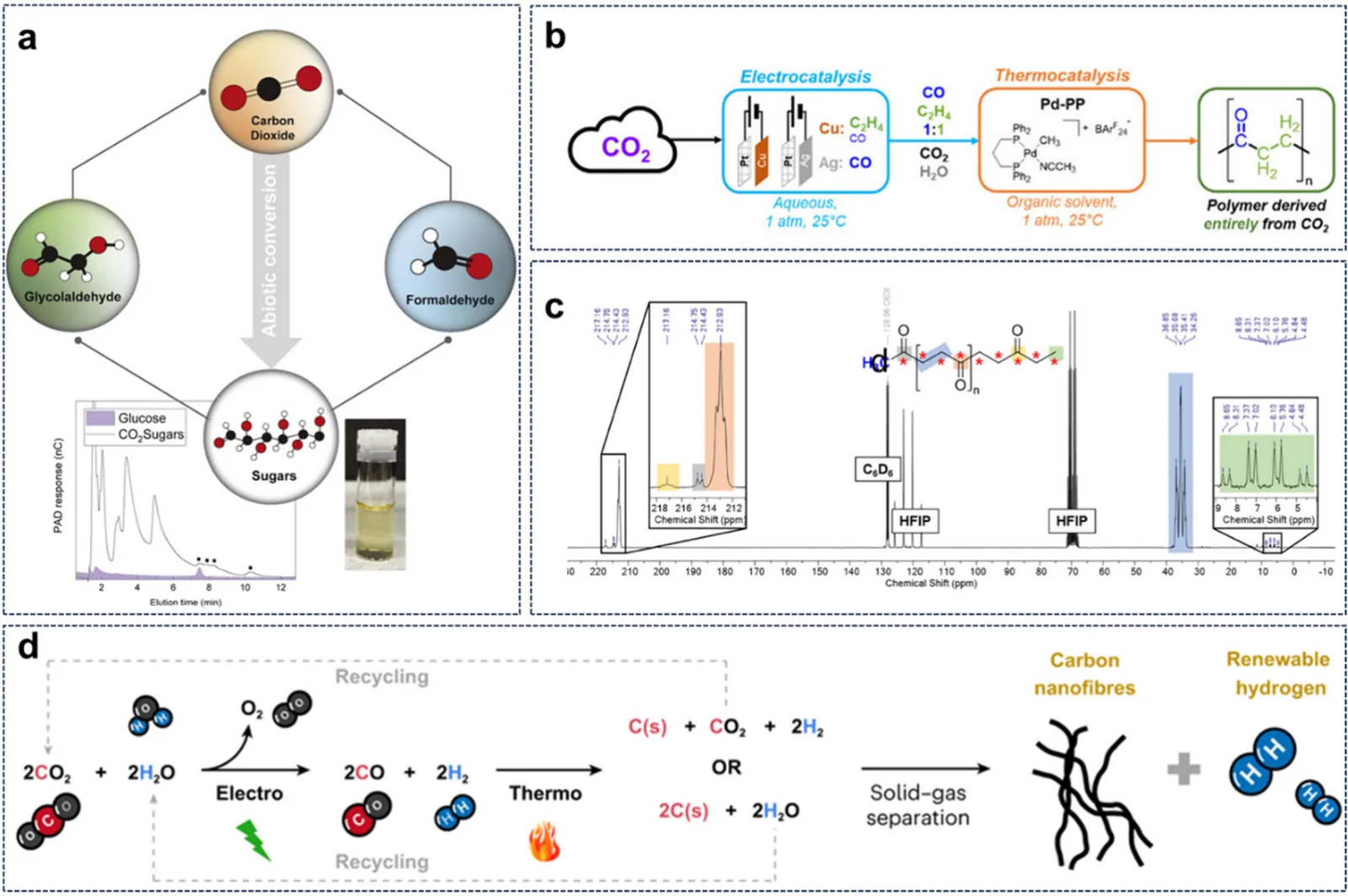
Electrocatalytic-thermocatalytic reaction for CO_2_ conversion. (a) Cascade conversion of CO_2_ to sugar, where CO_2_ is first electrochemically converted to formaldehyde and glycolaldehyde, followed by thermocatalytic process through Formose reaction to form sugars including glucose. Adapted with permission from ref 11. Copyright 2022 Elsevier Inc. (b) Schematic of cascade electrocatalytic-thermocatalytic system which couples electro-conversion (CO_2_ to CO and ethylene) and thermocatalytic CO-ethylene copolymerization to polyketones. (c) Analysis of ^13^C-labeled polyketone derived from ^13^CO_2_. Adapted with permission from ref 10. Copyright 2025 Wiley-VCH GmbH. (d) Scheme of the electrocatalytic-thermocatalytic tandem process of converting CO_2_ to carbon nanofibers. Adapted with permission from ref 31. Copyright 2024 Springer Nature.

Other long-chain chemicals, including C_3_ oxygenate,^29^ β-butyrolactone,^30^ and propionaldehyde^20^ have also been reported by the rational design of reaction routes and controlled selectivity. For instance, polyketones are crucial platform chemicals with excellent strength, elongation resistance, and environmentally friendly features. Zhelyabovskiy et al. reported a cascade electrocatalytic-thermocatalytic system, which coupled eCO_2_RR (to CO and ethylene) and thermocatalytic CO-ethylene copolymerization to form polyketones (Figure 3b,c).^10^ In this work, a Cu gas-diffusion-electrode (GDE) and a silver GDE were used in series to tune the equal concentrations of CO and C_2_H_4_ products, followed by a recirculatory CO_2_ reduction loop for achieving higher CO and ethylene concentrations. The Chen group reported an electrochemical-thermochemical tandem strategy for CO_2_ to carbon nanofibers (Figure 3d), during which the coelectrolysis of CO_2_ and water into syngas (CO and H_2_) avoided energy-intensive conditions, realizing a carbon nanofiber production rate of 2.5 g_carbon_ g_metals_^–1^ h^–1^ at relatively mild conditions (370–450 °C, 1 atm).^31^ Other industrial chemicals, including butane,^32^ ethylene oxide,^33^ and mixtures of benzene, toluene, ethylbenzene, and xylene isomers (BTEX),^34^ have also been produced by electrocatalytic-thermocatalytic cascade reactions using CO_2_ and other reactants, suggesting a broader role for CO_2_ electrolysis as a source of carbon feedstocks that can be further utilized to the existing chemical industry, rather than as a stand-alone route to the final products.

### 2.3 Electrocatalytic-Biocatalytic Reactions for CO_2_ Conversion

Biocatalytic reactions can be mainly classified into microorganism fermentation and enzymatic reactions.^35^ In the past several years, progress has been made on the efficient electrochemical CO_2_ conversion to C_1_ and C_2_ products (e.g., HCOOH, CH_3_COOH) with high selectivities, which can serve as food/energy input for microorganism fermentation or enzymatic reactions toward food production.^36–38^ Thus, integrating electrocatalytic reactions with biocatalytic reactions can provide efficient CO_2_ valorization under mild conditions. Although substantial efforts have been explored for directly attaching microbes on electrodes, it still suffers from low activities. Thus, spatially segmented electrocatalytic reaction and microorganism fermentation are adopted for achieving long-chain products.^39^ As for enzymatic reactions, the enzymes can be precisely regulated, where specific systems are designed for different products, benefiting the production of diverse multicarbon products.^40^

For example, Ma and co-workers reported a chemical-biochemical hybrid pathway for the CO_2_-to-starch production in a cell-free system, which inspires the possibility of artificial electro-biohybrid carbohydrate synthesis.^40^ Xiong and co-workers reported a hybrid electrocatalytic-biocatalytic flow system, which combined eCO_2_RR to formate and a subsequent five-enzyme cascade platform to achieve C_6_ sugars (Figure 4a).^16^ The Yang group presented a cascade abiotic–biotic system, which coupled electroconversion of CO_2_ to C_2_ oxygenates with biopolymerization to poly­(3-hydroxybutyrate) using C. *necator* in the bioreactor.^17^ A biocompatible electrolyte (0.1 M KH_2_PO_4_) replaces biotoxic alkali to resolve the biotoxicity issue. As the electro-synthesized products, such as acetic acid, can mix with concentrated electrolyte (e.g., KOH), energy-intensive separation and purification processes are often used to avoid rapid apoptosis of microorganisms.^41^ To tackle this challenge, Xia and co-workers reported an electrocatalytic-biocatalytic system that coupled a two-step CO_2_ electrolysis (CO_2_-to-CO and then CO-to-acetic acid) and yeast fermentation (Figure 4b,c).^41^ In this work, a porous solid-electrolyte reactor was employed to concentrate and collect the as-generated pure acetic acids, which minimizes energy and economic waste. In addition, the employment of genetically engineered microbes enables pure acetic acid as the growth condition and efficient *in vitro* production of glucose.

**Figure 4:**
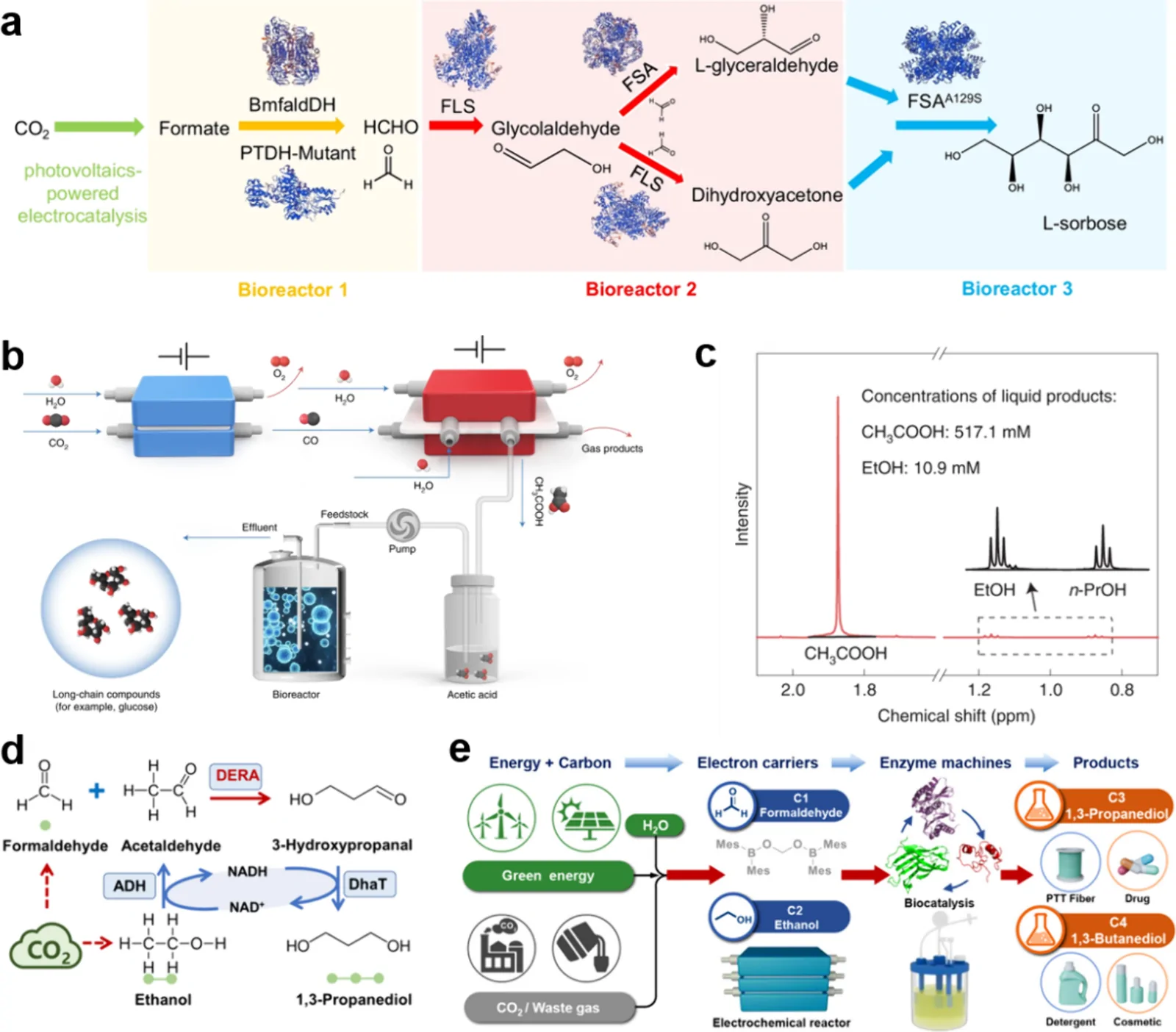
Electrocatalytic-biocatalytic reaction for CO_2_ conversion. (a) Synthetic pathways for converting CO_2_ to C_6_ sugar-l-sorbose. Reproduced from ref 16. Available under a Creative Commons CC BY license. Copyright 2024 Springer Nature. (b) Schematic illustration of the *in vitro* artificial sugar synthesis system, where CO_2_ was first converted to pure acetic acid through two-step electrolysis, and acetic acid was then directly fed for microorganism fermentation to produce long-chain compounds. (c) Product analysis showing that the CH_3_COOH concentration was significantly higher than that of other byproducts, revealing the high relative purity of acetic acid solutions. Adapted with permission from ref 41. Copyright 2022 Springer Nature. (d) Biocatalytic production pathway for 1,3-propanediol. (e) Schematic of the electrocatalytic-biological cascade system for CO_2_ conversion to 1,3-propanediol and 1,3-butanediol. Reproduced from ref 13. Copyright 2025 American Chemical Society.

Artificial-intelligence (AI) assisted biocatalysis has also been integrated with electrocatalysis in cascade systems for producing 1,3-propanediol and 1,3-butanediol,^13^ which are high-value chemicals in polymer synthesis, cosmetics, and pharmaceuticals. Using a CuZn alloy catalyst, CO_2_ was electrochemically converted into ethanol as the upstream product, which was subsequently coupled with formaldehyde to produce 1,3-propanediol and 1,3-butanediol at rates of 1.8 and 1.0 g L^–1^ h^–1^, respectively (Figure 4d,e).^13^ A double mutant combining the best mutation identified by AI was created in the rate-limiting enzyme Aldolase (DERA), further enhanced 1,3-propanediol production, showing how enzyme engineering can help convert a simple eCO_2_RR alcohol into higher-value products. In this scenario, CO_2_ electrolysis does not have to make the final molecule directly. Instead, it can provide biologically usable carbon feedstocks such as formate, acetate, and ethanol that microbial or enzymatic systems can upgrade into sugars, polymers, diols, and related products.

## 3 Challenges and Design Goals

Despite the progress outlined above, moving electrochemical cascade CO_2_ conversion from proof-of-concept demonstration to scalable synthesis still remains highly challenging. First and foremost, how those systems can be further developed to reach industrially relevant performance should be designed across the entire cascade. For electrochemical routes to become economically competitive, the electrical-to-chemical conversion efficiencies of at least 60% are generally considered necessary.^42^ Among currently accessible eCO_2_RR products, CO is the closest product toward technical readiness and has been demonstrated with Faradaic efficiencies exceeding 95% and ampere-level current densities.^43,44^ Nonetheless, CO is mainly an upstream intermediate rather than a final high-value product. For more complex cascade products, the production rates listed in Table 1 remain far below those required to displace established petrochemical processes. The central performance gap therefore exists not only in improving the upstream eCO_2_RR step but also in maintaining high current density, Faradaic efficiency, single-pass conversion, and energy efficiency throughout the entire cascade.

First and foremost, how those systems can be further developed to reach industrially relevant performance should be designed across the full cascade.

In addition, the precise control of catalysts to realize efficient formation and stabilization of reactive intermediates and/or upstream products at multiphase interfaces is still difficult, resulting in many possible side reactions and inefficient energy utilization. Another barrier is that a quantitative understanding of the coupled mass, charge, and energy-transfer processes remains incomplete. Although several *in situ*/operando multimodal spectroscopy techniques have been developed to capture reaction intermediates, the intermediates on specific microregions are still difficult to characterize.^45,46^ The mechanisms of those cascade reactions need to be further explored, especially for downstream thermo-/biocatalysis, to provide a clear pathway for future cascade systems to follow. Moreover, the differences between the operating conditions, for example, pH, electrolyte, and temperature, of upstream and downstream may lead to extra purification procedures and energy waste. Thus, a rational design to bridge the gaps is also needed for a more-compatible cascade system. Addressing those challenges will be beneficial for achieving programmable high-efficiency cascade transformations.

Facing those challenges, future research will have to increasingly focus on how to develop/improve the electrocascading systems in the competition with petrochemical processes and how to overcome the barriers mentioned above so that the overall electrocascading system can be insightfully comprehended and intelligently designed. First, as the manufacturing scale and mature industry for commodity chemicals present high barriers for a new technology to penetrate those markets, the targeted products of the electrocascading system need to be focused on high-value fine chemicals instead of commodity chemicals. In this case, the low carbon emission technique, the mild reaction condition, and the low cost of renewable energy may show the prominent superiority of the electrocascading system over the traditional industry. For example, electrocatalytic deuteration with D_2_O as the deuterium source has emerged as a mild and promising strategy for constructing key building blocks that are extensively utilized in synthetic chemistry and drug development,^47,48^ reducing the need for expensive deuterated reagents and harsh high-temperature reaction conditions in traditional syntheses.

Second, the AI toolkit/platform may become the next-generation solution for rational catalyst and enzyme design. The AI-assisted (bio)­catalyst designs may bypass the trial-and-error screening processes, which are not only resource-intensive but also often fail to capture correlations among variables, frequently yielding local rather than global performance optima.^49–51^ In addition, the detailed investigation using *in situ* or operando characterization techniques combined with theoretical modeling may provide new and deep insights into the mechanisms of the upstream eCO_2_RR reactions and the subsequent thermo-/biocatalysis. The innovation of microscale spectroscopy techniques may break the resolution bottleneck and provide a clear guide to comprehend the underlying mechanism.

As the manufacturing scale and mature industry for commodity chemicals present high barriers for a new technology to penetrate those markets, the targeted products of the electro-cascade system need to be focused on high-value fine chemicals instead of commodity chemicals.

Moreover, designing biocompatible electrolytes and membrane-free integration architectures can bridge electrochemical and biological catalysis, enabling artificial CO_2_ valorization systems that can be operated autonomously under renewable power. Some additional treatments should also be designed to tackle the incompatibility among different cascade modules. For example, the technology developed for carbon capture materials, including covalent organic frameworks (COFs), can be used for the separation of unreacted CO_2_ from products in the output stream.^52^ Optimizing single-pass conversion at high selectivity also shows promising potential in reducing separation costs downstream.^53^ Ultimately, realizing controllable CO_2_-to-molecule networks that emulate biological metabolism will mark a transformative milestone in sustainable chemistry.

## 4 Summary

The cascade electrocatalytic conversion of CO_2_ represents a paradigm shift in the pursuit of carbon-neutral chemical manufacturing. By integrating electrocatalytic, thermocatalytic, and biocatalytic processes within a unified framework, this approach transcends the limitations of conventional single-step CO_2_ reduction and enables stepwise molecular construction, flexible reaction control, and much-expanded product varieties, allowing the transformation of simple carbon feedstocks to complex and high-value chemicals. Recent examples of artificial CO_2_-to-sugar and CO_2_-to-polymer pathways have exemplified how cascade catalytic systems may unlock new capabilities of chemical synthesis and bridge renewable electricity to sustainable carbon utilization.

Potential strategies, including aiming at value-added products, leveraging AI to assist the catalysts design, evaluating mechanisms by integrating *in-situ*/operando spectroscopy and microscopy techniques, and rational design of compatible reactors and reaction conditions, may break the barriers.

Although many reported systems have suggested attractive potentials of utilizing CO_2_ as the primary carbon feedstock for electro-cascade reactions, the electro-cascade system still cannot displace the current industrial manufacturing due to many challenging bottlenecks, including the limited production scale, the imprecise fabrication of catalysts, the incomplete mechanism understanding, and the mismatched operating conditions between up-/downstream. Potential strategies, including aiming at value-added products, leveraging AI to assist the catalysts design, evaluating mechanisms by integrating *in situ*/operando spectroscopy and microscopy techniques, and rational design of compatible reactors and reaction conditions, may break those barriers. Looking into the future, cascade electrocatalysis not only provides a possible approach for CO_2_ utilization but also offers a pathway toward programmable chemical manufacturing driven by renewable energy—bringing the vision of artificial carbon cycle closer to reality.
